# Time-Resolved Spectroscopic Study on the Photoredox Reaction of 2-(*p*-Hydroxymethyl)phenylAnthraquinone

**DOI:** 10.1038/s41598-017-09192-3

**Published:** 2017-08-22

**Authors:** Qingqing Song, Xiting Zhang, Jiani Ma, Yan Guo, David Lee Phillips

**Affiliations:** 1 Key Laboratory of Synthetic and Natural Functional Molecule Chemistry of Ministry of Education, College of Chemistry and Materials Science, Northwest University, Xi’an, People’s Republic of China; 2 Department of Chemistry, The University of Hong Kong, Pokfulam Road, Hong Kong S.A.R., P. R. China

## Abstract

In this work, we report a combined time-resolved spectroscopic and density functional theory computational study on 2-(*p*-hydroxymethyl)phenylanthraquinone (PPAQ) in which the benzyl alcohol moiety is significantly farther away from the AQ ketone group compared to the compound 2-(1-hydroxyethyl) 9,10-anthraquinone (HEAQ) so as to investigate the photophysical and photochemical reactions of PPAQ in several solvents, especially for the photoredox reaction in a pH 2 aqueous solution. The results here indicate that PPAQ undergoes the photoredox reaction via a two-step pathway and that the low efficiency of the photoredox reaction of PPAQ compared to the related HEAQ molecule is caused by the longer distance between the benzyl alcohol moiety and the AQ ketone moieties.

## Introduction

Anthraquinone (AQ) is an important component of the anticancer drug anthracycline^1–3^ and AQ and its derivatives have also been widely studied for many types of photochemical reactions. Photoexcitation of AQ compounds often leads to efficient intersystem crossing (ISC) to populate a reactive triplet state that may then undergo a variety of reactions such as a typical hydrogen abstraction reaction with a strong hydrogen donor solvent like isopropanol (IPA)^4–6^. The photochemistry of AQ derivatives in aqueous solutions had also received increasing interest as a new platform for developing photoremovable protecting groups for a range of applications^7–13^.

An unusual photoredox reaction was discovered for AQ compounds by Wan and coworkers^14^. We recently examined 2-(1-hydroxyethyl) 9,10-anthraquinone (HEAQ) to study its photoredox reaction mechanism in an acidic aqueous solution using time-resolved spectroscopic experiments and density functional theory (DFT) calculations^15^. This work revealed that HEAQ undergoes an efficient photoredox reaction in water containing solutions via an initial protonation on the carbonyl oxygen, followed by a deprotonation of the side methylene C-H bond. Recently, a computational investigation using CASSCF calculations examined the reaction route of the photoredox reaction of HEAQ^16^. This study found that in a neutral aqueous solution, the photoredox reaction of HEAQ appears to occur via an excited state intramolecular proton transfer (ESIPT) process^16^. The photoredox reaction can be rationalized by excited states that have substantial charge transfer character, in which the electron density of the benzene ring with the reactive CH_2_OH moiety is transferred to the central AQ ring. The trapping by the carbonyl oxygen of a proton from water leads to the observed overall redox chemistry. Our recent experimental and theoretical results in neutral aqueous solutions indicate that a proton-coupled electron transfer from an alcohol C-H bond to the *para*-carbonyl is the initial and crucial process for the photoredox reaction of HEAQ to occur in neutral aqueous solutions, which is caused by an intriguing charge-radical coupled effect. This could account for the experimental results in the literature that HEAQ can undergo efficient photoredox reaction under neutral aqueous conditions^17^.

**Figure 1:**
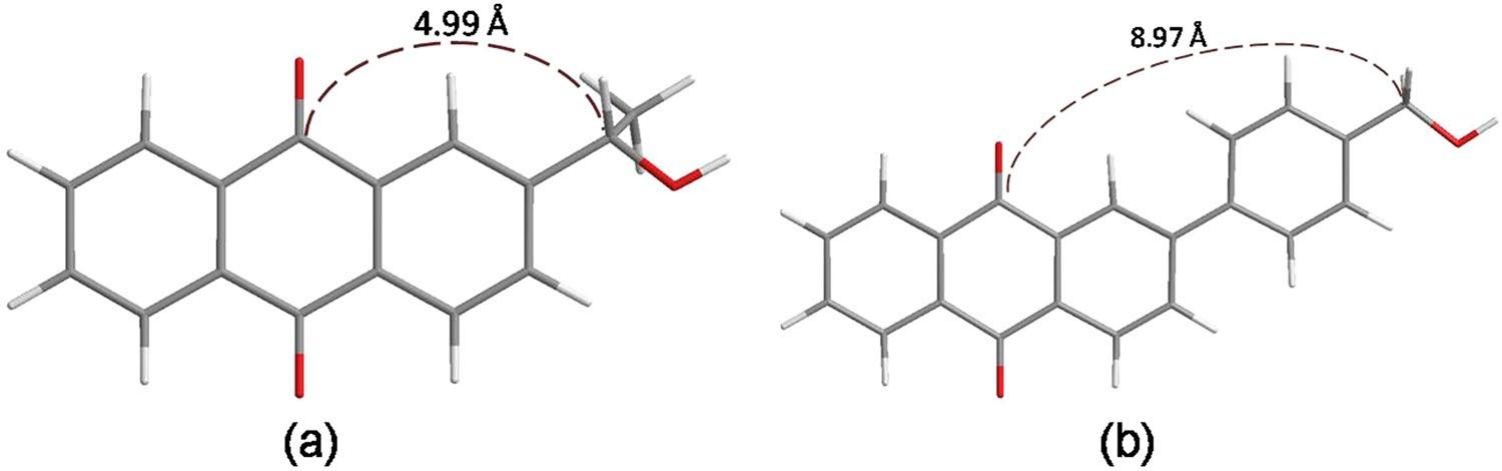
Optimized chemical structures of the triplet state of (**a**) HEAQ and (**b**) PPAQ obtained from B3LYP/6-311 G** DFT calculations.

**Figure 2:**
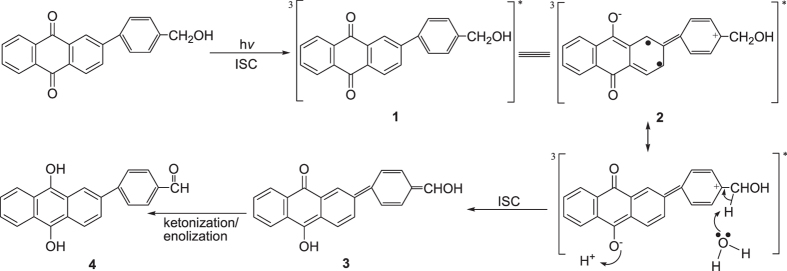
A proposed concerted reaction mechanism of the photoredox reaction of PPAQ in acid aqueous solutions based on that of ref. 18.

**Figure 3:**
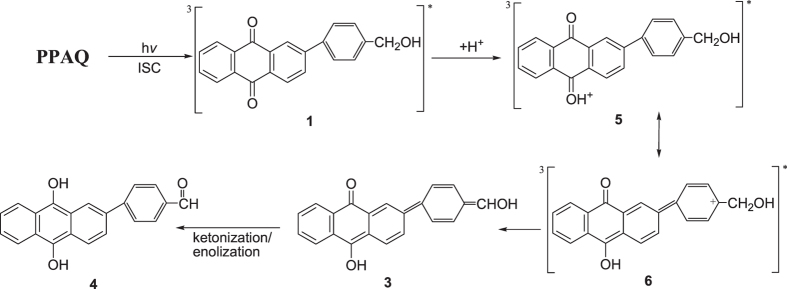
A proposed two-step reaction mechanism of the photoredox reaction of PPAQ in acidic aqueous solutions based on the results of a previously reported mechanism study on HEAQ is shown.

It is noteworthy that AQ compounds with a benzyl alcohol moiety much further away from the AQ ketone groups like 2-(*p*-hydroxymethyl)phenylanthraquinone (PPAQ) can also undergo a photoredox reaction in aqueous solutions with a fairly high quantum yield (*Φ* ~ 0.5, pH 1 MeCN-H_2_O) but not as high as was found for HEAQ under similar conditions^18^. Final product analysis results clearly demonstrated that the insertion of an additional phenyl group has modulated the reaction efficiency. It is important to know the modulation mechanism for the distance between the ketone group and the benzyl alcohol moiety (caused by the insertion of a phenyl ring between these two functional groups) on the photoredox reaction of AQ compounds. On one hand, it is suggested from the DFT calculations that the atomic distance between the carbonyl carbon atom and the ethyl carbon atom in the side chain is remarkably longer for the triplet state species of PPAQ (8.97 Å) than that for the triplet state species of HEAQ (4.99 Å), see Fig. 1. This brings up the question, how the long distance between the carbonyl carbon atom and ethyl carbon atom influences the photoredox reaction of AQ compounds? On the other hand, the insertion of the electron rich phenyl ring between the carbonyl and the ethyl group is expected to affect the electron transfer which plays a critical role in the overall photoredox reaction based on the results from our previous studies^15, 17^. Second, the intramolecular photoredox reaction in aqueous solutions for PPAQ was first proposed to take place through a concerted route (see Fig. 2) of an initial excited state electron migration from the aromatic ring of the benzyl alcohol to the AQ coupled by trapping with solvent protons^18^. This concerted reaction mechanism is different from the two-step route found in our previous time-resolved spectroscopic studies on HEAQ^15^. The proposed two-step reaction mechanism of PPAQ based on the study of HEAQ is depicted in Fig. 3. Do substrates containing the benzyl alcohol moiety far away from the AQ ketone group undergo the photoredox reaction in the same way as do substrates in which the benzyl alcohol moiety is close to the AQ ketone group? Third, it was found that HEAQ has a comparable quantum yield and reaction efficiency in a neutral aqueous solution with that in a moderate acidic condition, however, PPAQ performs very differently between neutral and moderate acidic aqueous conditions. The efficiency of the photoredox reaction of PPAQ was rather low at pH 7 from quantum yield measurements (*Φ* ~ 0.07) compared to HEAQ (*Φ* ~ 0.8, pH 7). Even in an acidic aqueous solution, PPAQ was found to be less reactive toward the photoredox reaction than HEAQ under analogous conditions. To help answer the above questions, we performed time-resolved spectroscopic studies on PPAQ using femtosecond transient absorption (fs-TA), nanosecond transient absorption (ns-TA) and nanosecond time-resolved resonance Raman (ns-TR^3^) spectroscopic techniques to study the photophysical and photochemical reactions of PPAQ in several solvents to compare with analogous results found previously for HEAQ. We also obtained results from DFT calculations to help interpret the time-resolved spectroscopy experimental results.

## Results and Discussion

### In Acetonitrile (MeCN) and IPA

**Figure 4:**
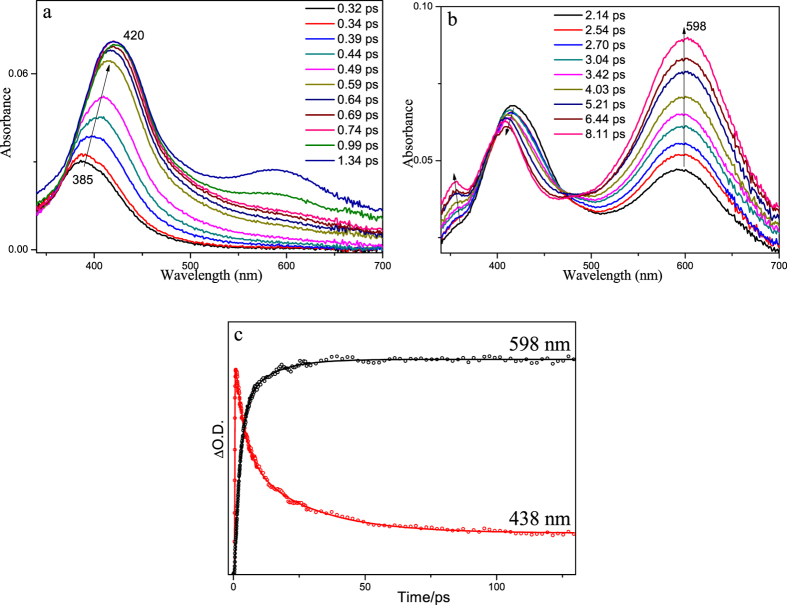
(**a** and **b**) The fs-TA spectra and (**c**) the temporal dependence of transient absorption at 438 and 598 nm for PPAQ in MeCN are shown.

**Figure 5:**
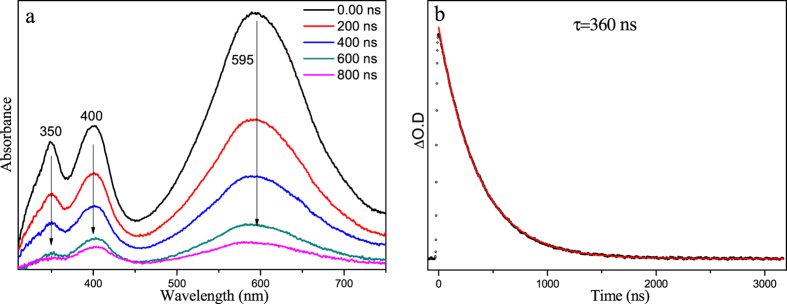
(**a**) The ns-TA spectra and (**b**) the temporal dependence of the transient absorption at 590 nm for PPAQ in MeCN are shown.

Figure 4 depicts the fs-TA results of PPAQ obtained in MeCN. At the very early time delays within 2 ps, the main band absorbing at 385 nm red-shifted to 420 nm. This change was also observed for HEAQ in MeCN at a comparable time region and can be assigned to the internal conversion (IC) process from a higher energy excited state (S_n_ (n > 1)) to the lowest singlet excited state (S_1_). The observation of clear isobestic points in Fig. 4b at 390 and 465 nm suggests a transformation of the S_1_ state to a new species. Because AQ compounds are known to commonly undergo efficient intersystem crossing (ISC) process to produce the triplet excited state species, the transformation observed in Fig. 4b is tentatively proposed to be the ISC process from S_1_ to the triplet state species (T_1_). To make further verification of this assignment, the ns-TA experiment was conducted (Fig. 5a) and the species with absorption bands at 350, 400 and 595 nm formed within the laser pulse. Kinetics analysis was conducted for the absorption at 590 nm and a lifetime of 360 ns was determined. The analogous spectral profile observed in ns-TA with that of the later species in fs-TA spectra suggests that the same species was observed in both the later time fs-TA and early time ns-TA spectra. The ns-TA spectra for PPAQ were also recorded in MeCN with saturated oxygen and the spectral profile keeps the same as that observed under an open air condition, while the lifetime of the species decreased to 120 ns. This behavior further supports that the species observed in the ns-TA spectra is the triplet excited state species of PPAQ (the triplet species is denoted as **1** hereafter) and the changes seen in Fig. 4b is due to the ISC process from S_1_ to T_1_. To obtain structural information regarding species **1**, ns-TR^3^ experiments were conducted for PPAQ in MeCN.

**Figure 6:**
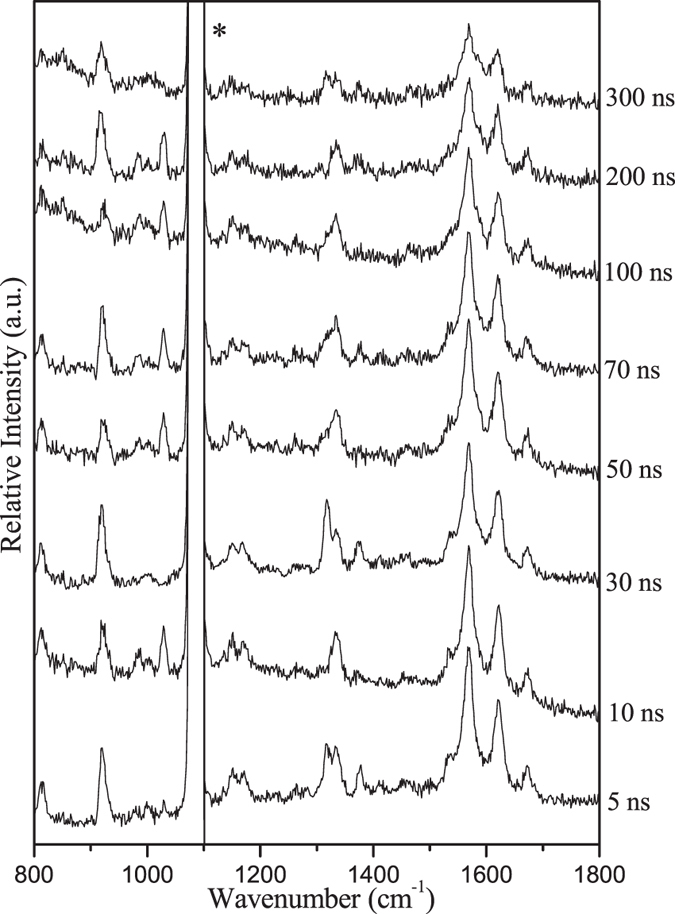
The ns-TR^3^ spectra of PPAQ after 266 nm photoexcitation in MeCN using a 416 nm probe wavelength at various time delays indicated next to the spectra are shown. The asterisk (*) marks regions affected by stray light.

Figure 1S displays the UV-vis spectrum of PPAQ in MeCN and the pump (266 nm) and the probe wavelengths (416 nm) used in the ns-TR^3^ experiment are indicated in the figure. Figure 6 show that mainly one species was observed upon irradiation and was assigned as **1** based on the fs-TA and ns-TA results obtained under analogous experimental conditions. Further support for this assignment comes from the comparison between the experimental results and the calculated normal Raman spectrum (Fig. 1S). These results suggest that PPAQ exhibits similar behaviors with that of HEAQ in MeCN, with the exception that the lifetime of **1** is remarkably shorter than that of the triplet state of HEAQ that had a time constant of 2000 ns for its lifetime under analogous conditions^15^.

**Figure 7:**
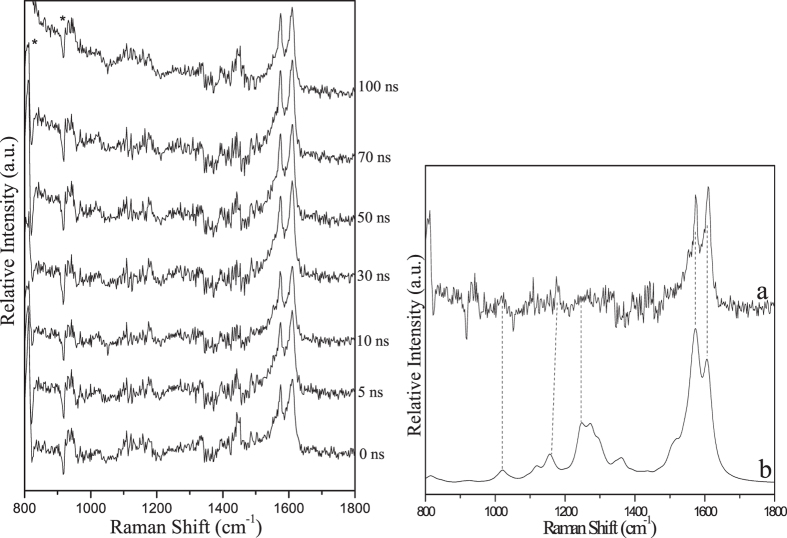
(Left) The ns-TR^3^ spectra of 2.0 × 10^−3^ M of PPAQ after 266 nm photoexcitation in IPA using a 416 nm probe wavelength at various time delays indicated next to the spectra are shown. The asterisk (*) marks regions affected by stray light. (Right) Comparison of (**a**) the experimental Raman spectrum obtained at 10 ns time delay of PPAQ in IPA to (**b**) the calculated normal Raman spectrum of the ketyl radical species of PPAQ.

**Figure 8:**
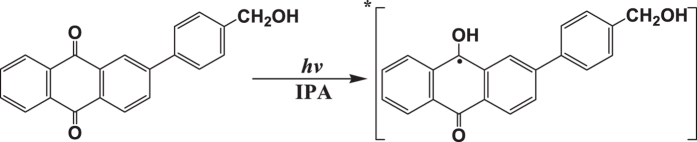
The photoreduction reaction of PPAQ in IPA.

Figure 7 (left) displays the ns-TR^3^ results for PPAQ in a strong hydrogen donor solvent IPA, where the typical photoreduction reaction of aromatic carbonyl compounds is expected to take place. The different Raman spectral profile in Fig. 7 (left) from that obtained in MeCN suggests that another species other than the species **1** was probed for PPAQ in IPA upon irradiation. Combining these results with the reported work on AQ compounds^4–6^ and especially the results for HEAQ in IPA^15, 17^, the new species detected in Fig. 7 (left) is thought to be the ketyl radical species of PPAQ, which is generated via abstraction of a hydrogen atom from the solvent IPA by the carbonyl oxygen group. Therefore, the experimental spectrum was compared with the calculated normal Raman spectrum of the ketyl radical species of PPAQ (see Fig. 7 (right)). The reasonable agreement between the experimental data and the calculated simulation further supports assignment of the species in ns-TR^3^ as being the ketyl radical species of PPAQ, and demonstrates that the main photochemical reaction for PPAQ in IPA is the photoreduction reaction via hydrogen abstraction (see Fig. 8). The detection of the ketyl radical species of PPAQ upon irradiation in ns-TR^3^ experiment suggests that the photoreduction reaction is very efficient for PPAQ in IPA, and this behavior was also observed for HEAQ^15, 17^.

### In Acidic MeCN-H_2_O Solutions

The above time-resolved spectroscopic studies revealed that PPAQ exhibits similar behaviors with HEAQ in both MeCN and IPA solutions. Time-resolved spectroscopic experiments were also conducted to study the photoredox reaction of PPAQ in acidic water-containing solutions.

**Figure 9:**
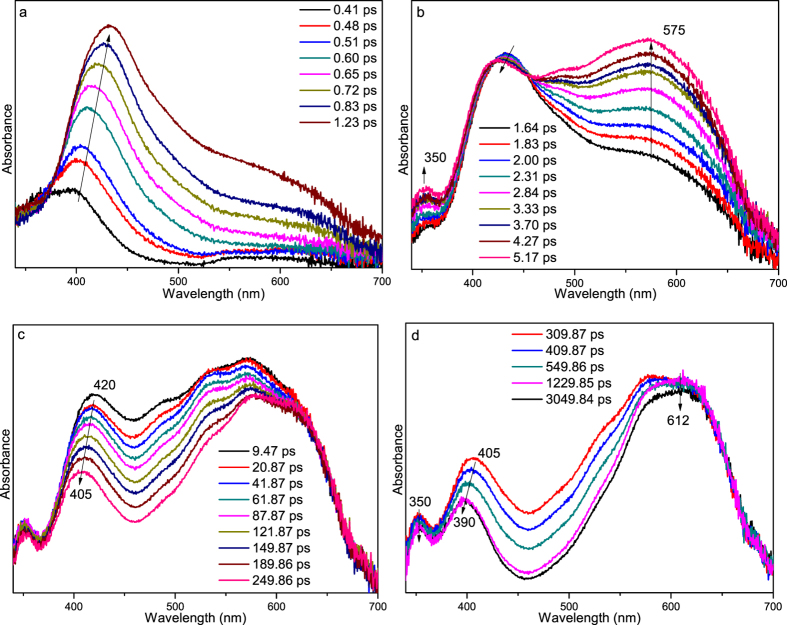
The fs-TA spectra of PPAQ in pH 2 MeCN-H_2_O (1:1) at different delay times recorded under 266 nm excitation are shown.

Figure 9 presents the fs-TA spectra of PPAQ obtained in an acidic aqueous solution. The steady state UV-vis spectra of PPAQ in MeCN, pH 2 MeCN-H_2_O (1:1) and pH 7 MeCN-H_2_O (1:1) solutions (see Figure 4S) shows no obvious difference, suggesting the original species upon irradiation in acidic aqueous solution is the same one as that in MeCN. Combining these results with the fs-TA results obtained in MeCN, the red-shift transformation from 395 nm to 432 nm within the first 1 ps (Fig. 9a) is assigned to be the IC process from S_n_ to S_1_ of PPAQ, followed by the ISC transformation producing the triplet species **1** with specific absorption bands at 350, 420 and 575 nm (Fig. 9b). It was noted that the spectral profile of **1** changed a bit compared to that in MeCN, which may be caused by the solvent effect since the PPAQ compound is easy to be affected by a polar solvent. The subsequent processes seem complicated. As a result, a new species was produced with characteristic absorption bands at 350, 390 and 612 nm, which was not detected in the ns-TA spectra obtained in MeCN. To make a clear demonstration, each fs-TA trace selected from Fig. 9a,b and c were presented together in Fig. 5S).

**Figure 10:**
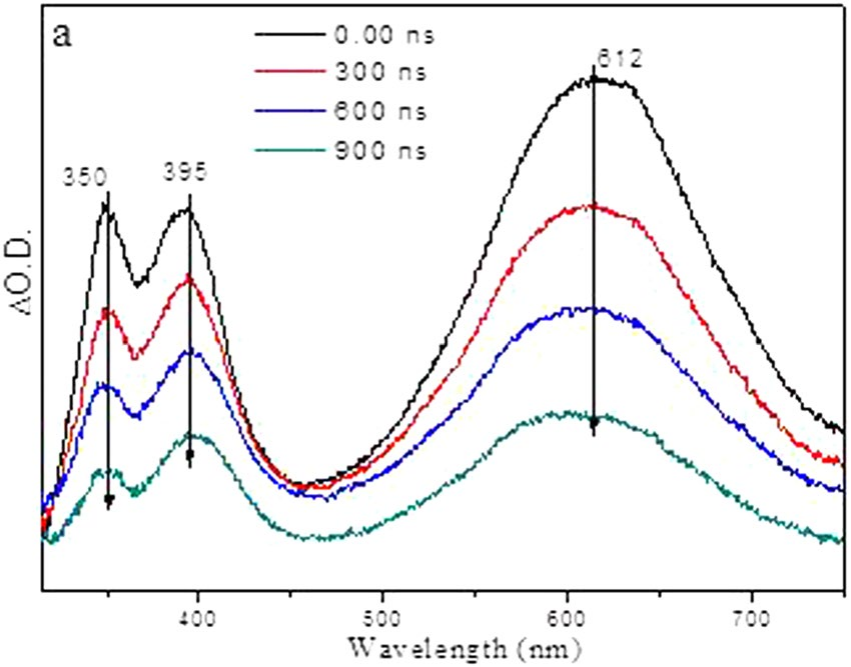
The ns-TA spectra of PPAQ in pH 2 MeCN-H_2_O (1:1) recorded with 266 nm excitation are shown.

**Figure 11:**
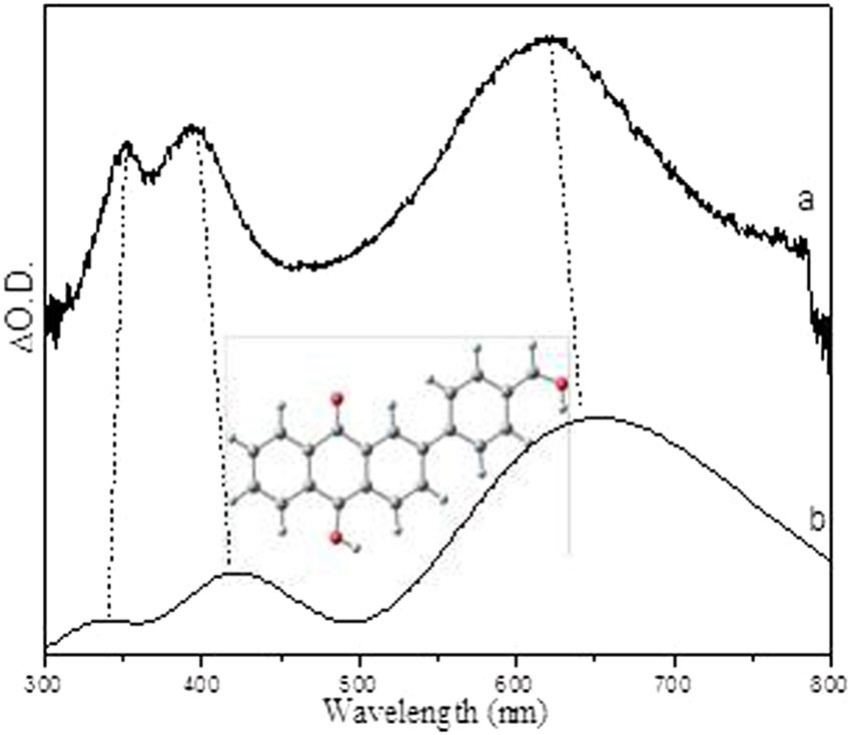
Comparison of (**a**) the experimental ns-TA spectrum of PPAQ obtained at 600 ns time delay of in pH 2 MeCN-H_2_O (1:1) recorded with 266 nm excitation to (**b**) the calculated UV-vis spectrum of the singlet state of the species **3** using um062x/6-311 G**.

To help the assignment of the species observed in the fs-TA experiment and to monitor the photochemical reaction of PPAQ in the subsequent time regions, ns-TA experiments were conducted for PPAQ in a pH 2 mixed aqueous solution and these results are displayed in Fig. 10. Mainly one species was observed in the ns-TA spectra and the spectral profile coincidences with the last species detected in the fs-TA spectra under analogous experimental conditions (Fig. 9d). The long lifetime of this new lygenerated species suggested it was probably a singlet state species. As the time-resolved spectroscopic experiments were conducted under the same conditions where the photoredox reaction was observed by Wan and coworkers, we therefore tentatively connected the generation of the new species with that involved in the photoredox reaction. Examination of Fig. 2 (**1** → **3** → **4**) and Fig. 3 (**1** → **5** → **3** → **4**) suggests that either in the concerted reaction route or the two-step reaction pathway, **1** generated the singlet species **3** which further underwent a ketonization process to form **4**. We therefore calculated the electronic spectra of **3** and **4** (See Fig. 11 and Fig. 7S) to compare with the experimental spectra, Clearly, the experimental spectra profile exhibited more similarity with the calculated spectrum for species **3**, rather than species **4**. Hence, it is proposed that species **3** was probed and directly observed in the fs-TA and ns-TA spectra. This assignment is consistent with Wan and coworkers study on a similar AQ compound in that the enol intermediate had a characteristic absorbance at 520 nm, and the decay of this species ultimately leads to a weak broad absorption in the 400 nm region and attributed to the photoredox product^18^.

**Figure 12:**
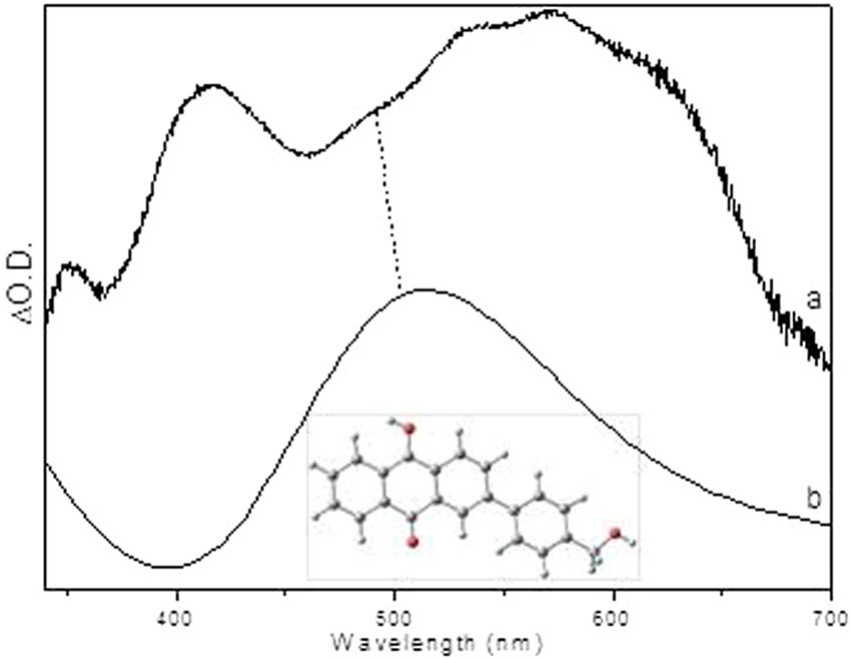
Comparison of (**a**) the experimental ns-TA spectrum of PPAQ obtained at 61.87 ps time delay of in pH 2 MeCN-H_2_O (1:1) recorded with 266 nm excitation to (**b**) the calculated UV-vis spectrum of the species **5** at *para*-postion using um062x/6-311 G**.

Closer examination of the fs-TA spectra especially in the time region between the observation of the species **1** and **3**, another species with an absorption band around 480 nm was generated. As in the concerted pathway mechanism shown in Fig. 2, the species that may possibly be observed between **1** and **3** is the triplet state species of **3**, namely **3** (0, 3). However, this possibility was excluded by the fact that the simulated UV-vis spectra for both the *meta*- and *para*-carbonyl **3** (0, 3) (shown in Fig. 6S) lack an absorbance feature near the region of 480 nm. On the other hand, the spectral profile shown in Fig. 9c exhibited great similarity with the calculated UV-vis spectra of the protonated species for both the *meta*- and *para*-carbonyl **5** (Fig. 12), which was proposed in the two-step pathway mechanism. It is therefore proposed that PPAQ mainly undergoes the photoredox reaction via the two-step pathway rather than the concerted pathway from the above results.

**Figure 13:**
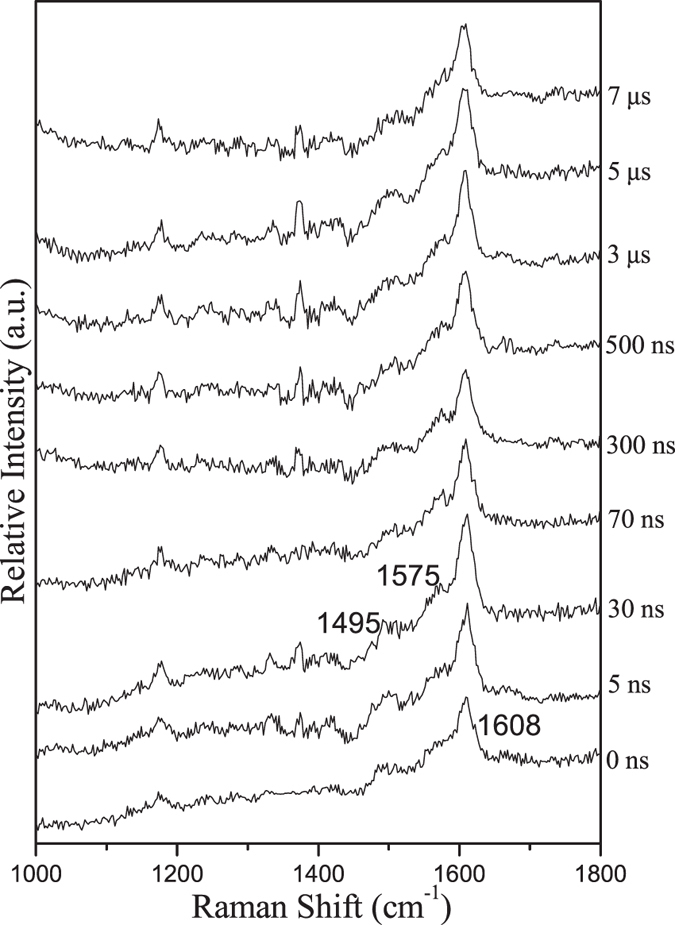
The ns-TR^3^ spectra of PPAQ after 266 nm photoexcitation in pH 2 MeCN-H_2_O (1:1) using a 355 nm probe wavelength at various time delays indicated next to the spectra are shown.

**Figure 14:**
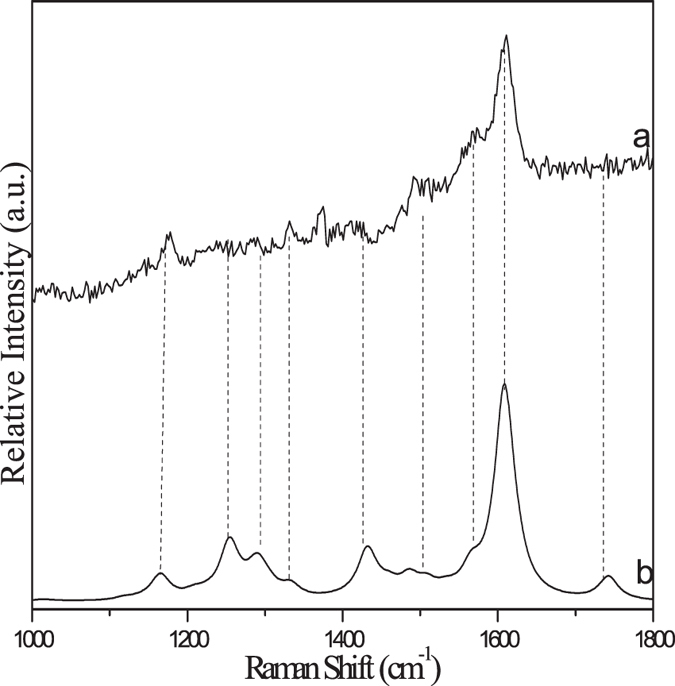
Shown is a comparison of (**a**) the experimental ns-TR^3^ spectrum obtained at 30 ns time delay of PPAQ in pH 2 MeCN-H_2_O (1:1) to (**b**) the calculated normal Raman spectrum of the species **4** using M062X/6-311 G**.

To gain structural information for the important intermediates, ns-TR^3^ experiments were conducted for PPAQ in a pH 2 mixed aqueous solution and these results are displayed in Fig. 13. It is found that mainly one species was observed with specific Raman bands at 1495, 1575 and 1608 cm^−1^ upon irradiation and lasts for a relatively long time. It is tentatively proposed that a ground state product species was detected here. Based on the above analysis of the transient absorption results, the experimental Raman spectrum of the species recorded at 30 ns was compared to the calculated Raman spectrum of the photoredox reaction product **4** (see Fig. 14). The good similarly between the experimental and calculated vibrational frequency pattern results supports that the species probed in the ns-TR^3^ spectra is species **4**. It is noted that species **3** was detected in the ns-TA spectra while species **4** was probed in the ns-TR^3^ experiments and no obvious signal from the species **3** was found. There are several possibilities that the species observed in ns-TA cannot be detected in the ns-TR^3^ experiment, the non-symmetrical structural, the low Raman scattering cross-section of the species and the low concentration of the species present. Firstly, the oscillator strength near the probe wavelength range of the 355 nm for species **4** (0.4428 at 343 nm) is substantially more intense than that of species **3** (0.1179 at 341 nm) as deduced from the electronic absorption calculations (shown in Fig. 11 and Fig. 5S). This suggests there is a high Raman scattering cross-section for species **4** since it gains significant Raman intensity by a stronger resonance with the transient absorption and is therefore observed in the ns-TR^3^ spectra more easily than species 3. Secondly, the more symmetrical structural of species **4** than that of species **3** facilitate the observation of the species **4** in our ns-TR^3^ experiments. Thirdly, the species **4** is much more stable than the species **3** (32 kcal/mol from DFT calculations). Based on the above facts, it is reasonable to propose that the totally symmetric vibrations of the species **4** which are resonant with the electronic transitions can be assumed to gain significant Raman intensity by resonance Raman enhancement. To sum up, we detected the key species involved in the photoredox reaction of PPAQ in a pH 2 aqueous solution by time-resolved spectroscopic experiments and with the assistance of results from DFT calculations we were able to determine that PPAQ undergoes the photoredox reaction via a two-step way as shown in Fig. 3.

### DFT Calculations

To better understand the photoredox reaction of PPAQ in aqueous solutions and explain its different behaviors from that of HEAQ, the spin and NBO analysis were performed by DFT calculations at the (U) M062X /6-311 G** level of theory and compared with analogous results for HEAQ.

**Figure 15:**
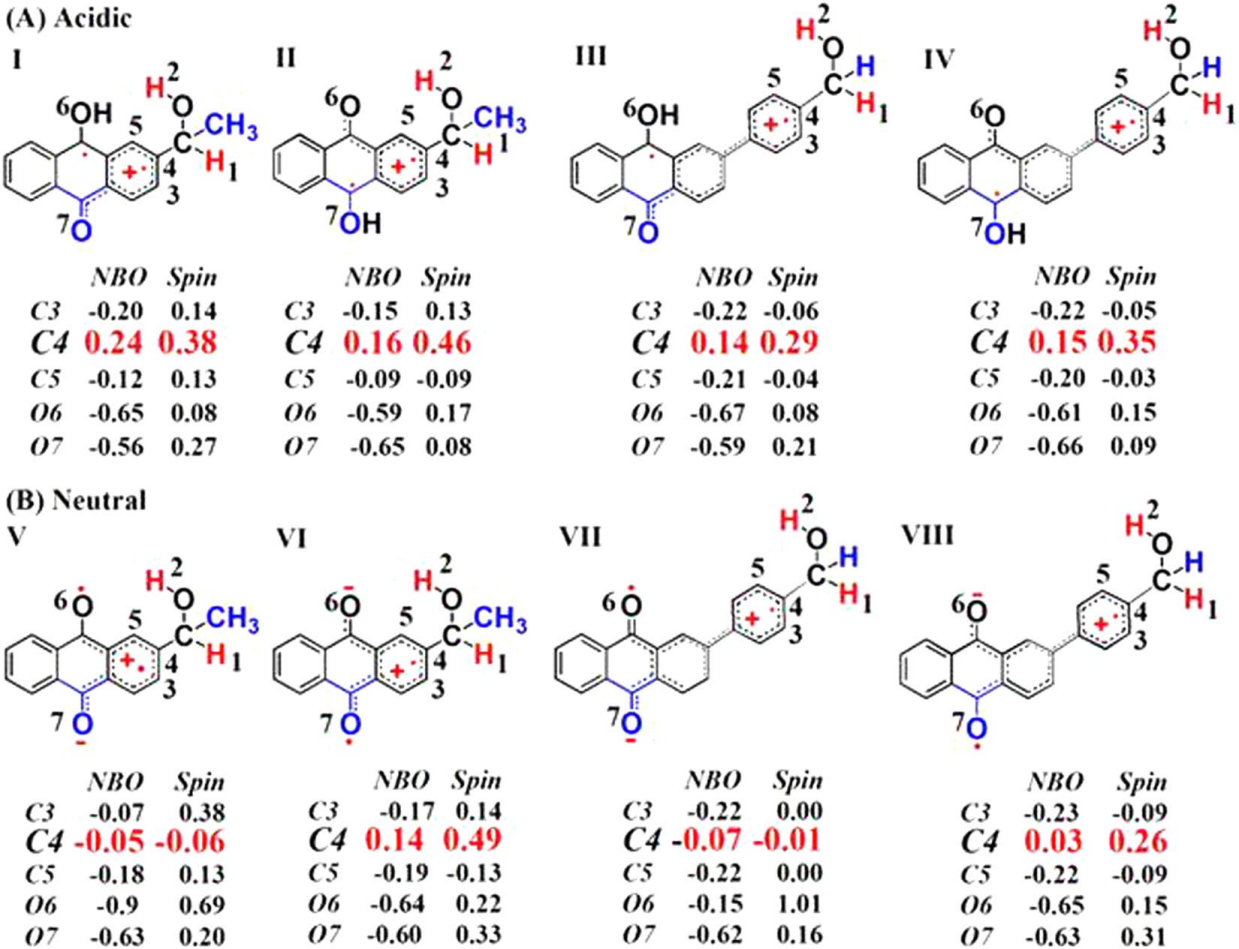
Shown are the NBO charge and spin density for HEAQ and PPAQ in acidic and neutral aqueous solutions.

The experimental results suggest that the efficiency of the photoredox reaction of PPAQ was rather low at pH 7 from quantum yield measurements (Φ ~ 0.07) compared to HEAQ (Φ ~ 0.8, pH 7) and PPAQ was found to be less reactive even in an acidic aqueous solution toward the photoredox reaction than HEAQ under analogous conditions. We try to account for these experimental phenomena. Why is PPAQ less reactive than HEAQ toward the photoredox at pH ≤ 7? It should be noted that the intramolecular redox reaction for 2-(hydroxymethyl)anthraquinone (HMAQ) also occurs with high efficiency (Φ ~ 0.8, pH 7), just like HEAQ presented here. HMAQ has the same substituent group (-CH_2_OH) as PPAQ. Therefore, the difference in the efficiency of the photoredox reaction is predominantly derived from the presence of a phenyl ring in PPAQ. The phenyl ring has a profound effect on the stability of the triplet π-π* state. The NBO charges and spin populations for HEAQ and PPAQ in acidic and neutral aqueous solution are displayed in Fig. 13. Our recent study demonstrated that the substituted phenyl and either the *meta*- or *para*-carbonyl can be excited in the π-π* triplets and the excited phenyl group may have an effect on the side chain^17^. The NBO charge and spin populated on the C4 atom, which is directly connected to the side chain, can be expected to play a decisive role on the cleavage of the side-chain C-H bond. It should be noted that AQs have *para*- and *meta*-carbonyl groups relative to the distal substituent group, leading to *meta*-protonation structures I and III and *para*-protonation structures II and IV, and excited *meta*-carbonyl neutral structures V and VII and excited *para*-carbonyl neutral structures VI and VIII. Figure 15 displays that the positive charge populated on C4 of PPAQ (0.15) is less than that of HEAQ (0.24) in acidic aqueous conditions. More positive charge in C4 will favor the proton released from the alcohol C-H bond of distal side chain. As a result, the alcohol carbon anion generated after the deprotonation process of the C-H bond of PPAQ cannot be stabilized by the positive charge on C4 as much as that in HEAQ, which is in good agreement with the experimental determination that HEAQ is more active toward the photoredox reaction than that of PPAQ. In neutral aqueous conditions, the positive charge is located on the C4 atom for both PPAQ and HEAQ (0.03 and 0.14, respectively) with the *para*-carbonyl group excited. The positive charge on the C4 atom for PPAQ is not positive enough to activate the alcohol C-H bond as much for the photoredox reaction compared to HEAQ. On the other hand, the spin and negative charge on the *para*-carbonyl O7 atom is an alternative point which can assist with pulling the proton transfer from the C-H cleavage. However, it is nearly the same in both HEAQ (charge: −0.60; spin: 0.33) and PPAQ (charge: −0.63; spin: 0.31). Therefore, the nearly absent photoredox reaction observed for PPAQ in neutral aqueous condition is mainly due to the poor positive charge population on the C4 atom resulting from the long distance between the carbonyl group and the side-chain group. The phenyl ring in protonation structures III or excited *para*-carbonyl neutral species VIII has nearly co-planar relationship with AQ moiety, which is seemed to favor the radical or charge delocalization to the distal substituent group at first glance. However, the NBO and spin distribution in Fig. 13 indicate that some spin or charge stay in the extra phenyl ring so that the spin and charge is significantly less delocalized to the C4 atom in III and VIII as compared with the C4 atom in I and VI, respectively. The extra phenyl ring in PPAQ acts as an electron pond so that the spin and charge cannot be efficiently transferred to the C4 atom, which significantly reduces the proton extrusion from the alcohol C-H bond of side chain. On the other hand, the long distance between carbonyl group and distal side chain require more water molecules to form a water bridge or hydrogen bond network connection for the hydrogen/proton transfer. The long water wire also will decrease the efficiency for the photoredox reaction for PPAQ as compared with the case in HEAQ. Overall, the phenyl ring reduces the efficiency of the electron transfer between carbonyl group and the C4 atom and long distance between carbonyl group and distal side chain decreases the efficiency of the proton/hydrogen transfer. As a result, the photoredox reaction for PPAQ is less efficient than that of HEAQ in both neutral and acidic aqueous solutions.

## Concluding remarks

**Figure 16:**
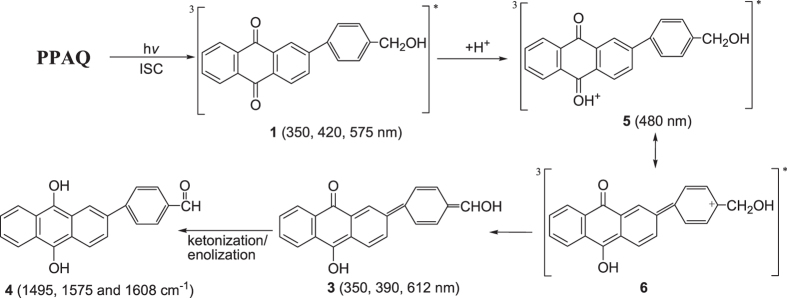
The proposed reaction mechanism of the photoredox reaction of PPAQ in aqueous solution. The letters and numbers below the structures are the corresponding labels used in the text. And the absorbance for the transient species detected from fs-TA and/or ns-TA spectra and the Raman frequency from the ns-TR^3^ are given.

Time-resolved spectroscopic experiments and DFT calculations were conducted to investigate the photoredox reaction mechanism for 2-(*p*-hydroxymethyl)phenyl anthraquinone (PPAQ) where the benzyl alcohol moiety is far away from the AQ ketone group, compared to a previously studied compound HEAQ. In acetonitrile, PPAQ undergoes an efficient intersystem crossing process to generate the triplet excited state species, **1**. In a strong hydrogen donor solvent IPA, **1** was quickly quenched by a hydrogen abstraction reaction and the ketyl radical species was detected in ns-TR^3^ spectra. In a pH 2 acidic aqueous solution, **1** was quenched via protonation on the carbonyl oxygen of the AQ group. Combining the results from the time-resolved spectroscopic studies, it is proposed that PPAQ undergoes the photoredox reaction via a two-step pathway (Fig. 16) in acidic aqueous solution in a manner similar to that previously reported for HEAQ.

The less efficient photoredox of PPAQ compared to that of HEAQ is caused by the long distance between the carbonyl group and the side-chain group as revealed by the spin and NBO analysis results from DFT computations. The work reported here can help in the development of the photoredox reaction for use in applications in the photosynthetic area and provides essential information for the use of AQ compounds in other photochemical applications such as a new platform for developing photoremovable protecting groups.

## Experimental and Computational Methods

### Femtosecond Transient Absorption (fs-TA) Experiments

The fs-TA measurements were performed using a regenerative amplified Ti:Sapphire laser system and an automated data acquisition system. The probe pulse was obtained by using approximately 5% of the amplified 800 nm output from the laser system to generate a white-light continuum (350–800 nm) in a CaF_2_ crystal. The maximum extent of the temporal delay was 3300 ps for the optical stage used in the experiments. The instrument response function was determined to be about 150 fs. At each temporal delay, data were averaged for 2 s. The probe beam was split into two parts before passing through the sample. One portion of the probe beam travels through the sample, the other portion was sent directly to a reference spectrometer that monitored the fluctuations in the probe beam intensity. Fiber optics were coupled to a multichannel spectrometer with a CMOS sensor that had a 1.5 nm intrinsic resolution. For the present experiments, the sample solutions were excited by a 266 nm pump beam (the third harmonic of the fundamental 800 nm from the regenerative amplifier). The 40 ml sample solutions were flowed through a 2 mm path-length cuvette throughout the data acquisition. The samples were monitored for degradation by employing UV absorption spectroscopy during the measurements and replaced with fresh samples as needed to avoid noticeable sample degradation effects on the data collected. The data were stored as three-dimensional wavelength-time-absorbance matrices that were exported for use with a fitting software. The sample solutions for the fs-TA experiments were prepared to have an absorbance of 1 at 266 nm so as to have the same number of photons being absorbed for the same irradiating conditions in each case^19^.

### Nanosecond Transient Absorption (ns-TA) Experiments

The ns-TA measurements were carried out on a commercial laser flash photoexcitation setup. The 266 nm pump laser pulse was obtained from the fourth harmonic output of an Nd:YAG laser and the probe light was provided by a 450 W Xenon lamp. The sample was excited by the pump laser and at a right angle the probe light from the Xenon lamp was passed through the sample. The two beams were focused onto a 1 cm quartz cell. The transmitted probe light was then measured either by a single detector (for kinetic analysis) or by an array detector (for spectral analysis). The changes in the transmission properties were normally converted into changes of optical density (ΔOD). The signals analyzed by a monochromator were detected by a photomultiplier and the signal processed via an interfaced PC and analytical software. Unless indicated the sample solutions used in the ns-TA experiments were prepared with an absorbance of 1 at 266 nm.

### Nanosecond Time-Resolved Resonance Raman (ns-TR^3^) Experiments

The ns-TR^3^ experiments were performed using an experimental apparatus and methods described previously in our laboratory and only a brief account is given here^20^. The 266 nm pump wavelength supplied by the fourth harmonic of a Nd:YAG nanosecond pulsed laser and the 355 nm probe wavelength supplied by the third harmonic of a Nd:YAG laser, the 416 nm probe wavelength supplied by the first Stokes hydrogen Raman-shifted laser line from the third harmonic of a Nd:YAG laser were used in the ns-TR^3^ experiments. The pump pulse excited the sample to initiate the photochemical reactions and the probe pulse interrogated the sample and the intermediate species produced by the pump pulse. The laser beams were lightly focused and aligned so that they were overlapped onto a flowing liquid stream of sample. The diameter of the pump beam was adjusted to be slightly larger than that of the probe beam at the overlapping volume in the liquid jet in order to minimize the ground state normal Raman signal. A pulse delay generator was used to electronically control the time delay between the pump and probe laser beams from the two different Nd:YAG lasers operated at a repetition rate of 10 Hz. The Raman scattered light was acquired using a backscattering geometry and then detected by a liquid nitrogen-cooled charge-coupled device (CCD) detector. The TR^3^ signal was acquired for 10 s by the CCD before being read out to an interfaced PC computer and 10 scans of the signal were accumulated to produce a resonance Raman spectrum. The ns-TR^3^ spectra presented in this report were obtained from subtraction of an appropriately scaled probe-before-pump spectrum from the corresponding pump-probe resonance Raman spectrum to remove non-transient bands. The Raman bands of MeCN were employed to calibrate the Raman shifts of the Raman spectra with an estimated accuracy of 5 cm^−1^.

### Density Functional Theory (DFT) Calculations

Density functional theory (DFT) calculations with the M062X level of theory at 6-311 + g** basis set in the solvent with SMD (MeCN) appears to be reliable to simulate the photochemical behavior for main-group compounds^17, 21–23^. The M06-2X functional performs well for the hydrogen-transfer barrier height calculations, proton affinities of conjugated systems, hydrogen bonding, electronic excitation, thermochemistry, kinetics and noncovalent interactions for the main-group compounds^20^. Our previous study on benzophenone derivatives revealed that the solvent polarity and the hydrogen interaction have significant effects on the stabilization for the excited triplet ππ* state and proton coupled electron transfer pathway compared to the excited triplet nπ* state and hydrogen atom transfer pathway (see Fig. 7S in the Supporting Information). Water molecules were considered as the explicit solvent while MeCN as the implicit solvent to simulate the mixed MeCN-H_2_O solutions in the current work. Therefore, the (U) M062X method with a 6-311 + G** basis set in SMD (MeCN) were done to determine the optimized geometries and vibrational wavenumbers for the species that were considered to be potential intermediates. The Raman spectra were obtained using the default G09 method that utilized the determination of the Raman intensities from the transition polarizabilities calculated by a numerical differentiation and with an assumed zero excitation frequency (e.g. the Placzek approximation). A Lorentzian function was used for the Raman vibrational frequencies and the relative intensities to obtain the computed Raman spectra that were compared to the experimental TR^3^ spectra. Appropriate frequency scaling factor of 0.983 was used in the comparison of the calculated results with the experimental spectra. No imaginary frequency modes were observed at the stationary states of the optimized structures shown here and only one imaginary frequency was observed for the saddle point transition state structures. The calculated UV-vis spectrums were carried out with the scaled value of 1.08 for X axis. The calculations presented in this study were done using the Gaussian 09 program suite installed on the High Performance Computing cluster at the Computer Centre in The University of Hong Kong.

## Electronic supplementary material

Supplementary Information

## References

[1] Guin PS, Das S, Mandal PC (2011). Interaction of 1,4-dihydroxy-9,10-anthraquinone with calf thymus DNA: a comparison with anthracycline anticancer drugs. J. Solution. Chem..

[2] Li, N. *et al*. Interaction of anticancer drug mitoxantrone with DNA analyzed by electrochemical and spectroscopic methods. *Biophys. Chem.* **116**, 199–205 (2005). 10.1016/j.bpc.2005.04.009 15893412

[3] Yang L (2015). Probing the interaction of anthraquinone with DNA by spectroscopy, molecular modeling and cancer cell imaging technique. Chemico-Biological Interactions..

[4] Wikinson F (1962). Transfer of triplet state energy and the chemistry of excited states. J. Phys. Chem..

[5] Görner H (2003). Photoreduction of 9, 10-anthraquinone derivatives: transient spectroscopy and effects of alcohols and amines on reactivity in solution. Photochem. Photobiol..

[6] Tickle K, Wilkinson F (1965). Photoreduction of anthraquinone in isopropanol. Trans. Faraday Soc..

[7] Furuta T, Torigai H, Sugimoto M, Iwamura M (1995). Photochemical properties of new photolabile cAMP derivatives in a physiological saline solution. J. Org. Chem..

[8] Furuta T, Hirayama Y, Iwamura M (2001). Anthraquinon-2-ylmethoxycarbonyl (aqmoc):  a new photochemically removable protecting group for alcohols. Org. Lett..

[9] Ren M-G, Bi N-M, Mao M, Song Q-H (2009). 2-(1′-Hydroxyethyl)-anthraquinone as a photolabile protecting group for carboxylic acids. Photochem. Photobiol A: Chem..

[10] Brinson RG, Jones P (2004). B.Cagedtrans-4-hydroxy-2-nonenal. Org. Lett..

[11] Sarma SJ, Jones PBJ (2010). Photochemistry of 1,n-dibenzyloxy-9,10-anthraquinones. Org. Chem..

[12] Jones PB, Brinson RG, Sarma SJ, Elkazaz S (2008). Observation of heavy atom effects in the development of water soluble caged 4-hydroxy-trans-2-nonenal. Org. Biomol. Chem..

[13] Brinson RG, Hubbard SC, Zuidema DR, Jones PBJ (2005). Two new anthraquinone photoreactions. Photochem. Photobiol. A: Chem..

[14] Hou Y, Wan P (2008). Formal intramolecular photoredox chemistry of anthraquinones in aqueous solution: photodeprotection for alcohols, aldehydes and ketones. Photochem. Photobiol. Sci..

[15] Ma J (2012). How and when does an unusual and efficient photoredox reaction of 2-(1-hydroxyethyl) 9,10-anthraquinone occur? A combined time-resolved spectroscopic and DFT study. J. Am. Chem. Soc..

[16] Dai J (2015). Water-assisted self-photoredox of 2-(1-hydroxyethyl)-9,10-anthraquinone through a triplet excited stateintra-molecular proton transfer pathway. Phys. Chem. Chem. Phys..

[17] Zhang X, Ma J, Phillips DL (2016). To photoredox or not in neutral aqueous solutions for selected benzophenone and anthraquinone derivatives. J. Phys. Chem. Lett..

[18] Hou Y, Huck LA, Wan P (2009). Long-range intramolecular photoredox reaction via coupled charge and proton transfer of triplet excited anthraquinones mediated by water. Photochem. Photobiol. Sci..

[19] Kochevar, I. E., Redmond, R. W. In: Abelson, J. N.; Simon, M. I. (Eds), Methods in enzymology, 319, Academic Press, USA, 2000, 20. 10.1016/s0076-6879(00)19004-4 10907495

[20] Ma C (2006). Ultrafast time-resolved transient absorption and resonance raman spectroscopy study of the photodeprotection and rearrangement reactions of *p*-hydroxyphenacyl caged phosphates. J. Am. Chem. Soc..

[21] Zhao Y, Schultz NE, Truhlar DG (2006). Design of density functionals by combining the method of constraint satisfaction with parametrization for thermochemistry, thermochemical kinetics, and noncovalent interactions. J. Chem. Theory Comput..

[22] Zhang X (2016). Ketyl radical formation via proton-coupled electron transfer in aqueous solution versus hydrogen atom transfer in isopropanol after photoexcitation of aromatic carbonyl compounds. J. Org. Chem..

[23] Harshan AK (2015). Proton-coupled electron transfer: moving together and charging forward. J. Am. Chem. Soc..

