# Supplementary material for: Time-Resolved Spectroscopic Study on the Photoredox Reaction of 2-(p-Hydroxymethyl)phenylAnthraquinone
Source: Sci Rep. 2017 Aug 22;7:9154. doi: 10.1038/s41598-017-09192-3 (PMC5567363; doi:10.1038/s41598-017-09192-3)
Supplement: Supplementary file 1 — Supplementary Information [file 41598_2017_9192_MOESM1_ESM.pdf]

Supporting Information

Title: Time-Resolved Spectroscopic Study on the Photoredox Reaction of 2-(*p*-Hydroxymethyl)phenylAnthraquinone

***Authors**: Qingqing Song†[&], Xiting Zhang‡[&], Jiani Ma*† Guo Yan‡ and David Lee Phillips*‡*

Table of Contents

Graphic **Page**

Figure 1S.UV-Vis absorption spectra of PPAQ in MeCN. The pump and probe wavelengths used in ns-TR^3 measurements are indicated. **S2**

Figure 2S. Comparison of the experimental ns-TR^3 spectrum of the first species observed in MeCN at 5 ns time delay (top) to the calculated normal Raman spectrum of **1** (bottom). **S2**

Figure 3S. The steady state UV-vis spectra of PPAQ in MeCN, pH 2 MeCN-H_2O (1:1) and pH 7 MeCN-H_2O (1:1). **S2**

Figure 4S. Comparison of the fs-TA spectra of PPAQ obtained at 4.27 ps, 61.87 ps and 1229.85 ps time delay in pH 2 MeCN-H_2O (1:1) recorded with 266 nm excitation. **S3**

Figure 5S. The calculated UV-vis spectrum of the singlet state of the species **4** using um062x/6-311G**. **S3**

Figure 6S. The calculated UV-vis spectrum of the triplet state of species **3** using um062x/6-311G**. **S3**

Figure 7S. The intrinsic reaction coordinate of benzophenone in H_2O reveals both HAT and PCET directly connected to the same ketyl radical ArPKxw. **S4**

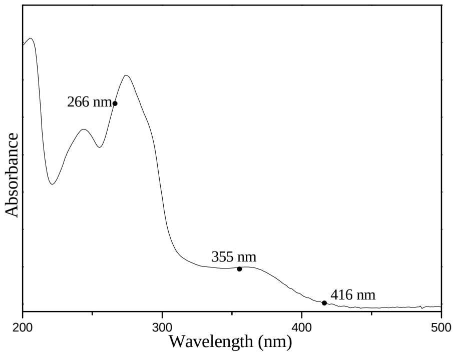

Figure 1S. UV-Vis absorption spectra of PPAQ in MeCN is shown. The pump and probe wavelengths used in ns-TR^3 measurements are indicated in the figure.

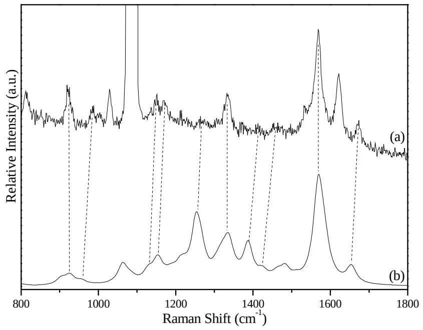

Figure 2S. Comparison of (a) the experimental Raman spectrum obtained at 10 ns time delay of 2.0×10^{-3} M of PPAQ in MeCN to (b) the calculated normal Raman spectrum of **1**.

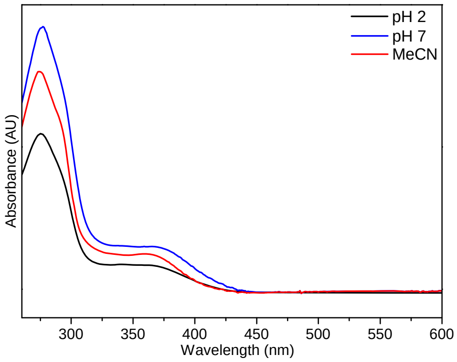

Figure 3S. The steady state UV-vis spectra of PPAQ in MeCN, pH 2 MeCN-H_2O (1:1) and pH 7 MeCN-H_2O (1:1).

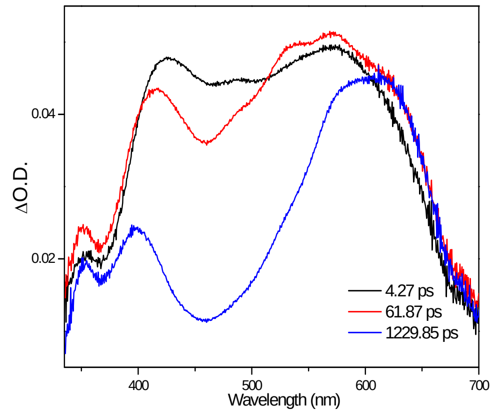

Figure 4S. Comparison of the fs-TA spectra of PPAQ obtained at 4.27 ps, 61.87 ps and 1229.85 ps time delay in pH 2 MeCN-H_2O (1:1) recorded with 266 nm excitation.

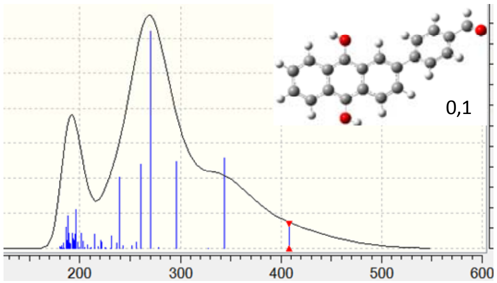

Figure 5S. The calculated UV-vis spectrum of the singlet state of the species **4** using um062x/6-311G**.

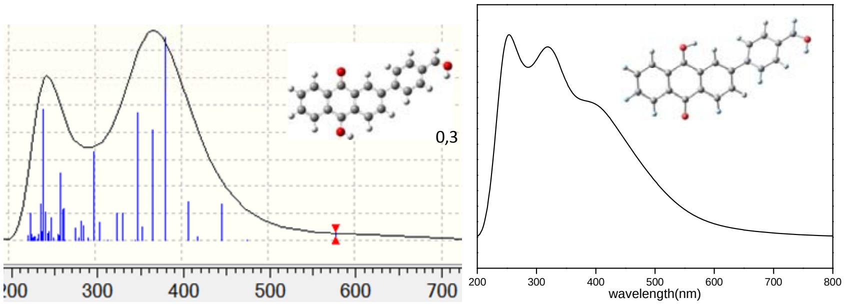

Figure 6S. The calculated UV-vis spectrum of the triplet state of the species **3** using um062x/6-311G**.

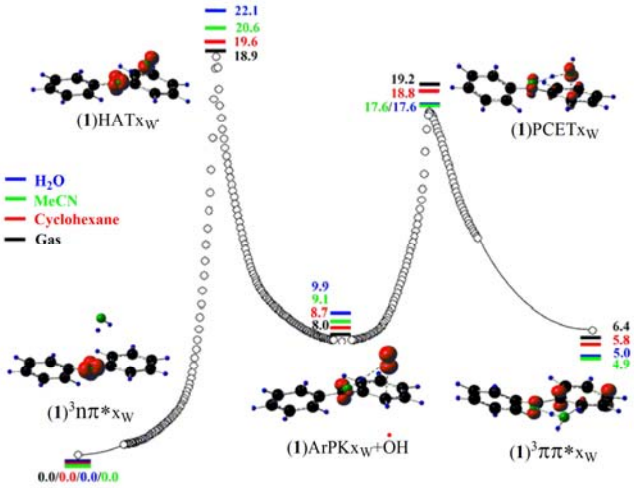

Figure 7S. The intrinsic reaction coordinate of benzophenone in H_2O reveals both HAT and PCET directly connected to the same ketyl radical ArPKxw.

Cartesian coordinates for the optimized geometry from the (U)B3LYP/6-311G** calculations for the species considered in this paper are given.

1

Center Number	Atomic Number	Atomic Type	Coordinates (Angstroms) X	Y	Z
1	6	0	6.290246	-0.261734	0.112184
2	6	0	5.342481	0.742957	0.058086
3	6	0	3.978073	0.427497	0.030121
4	6	0	3.581534	-0.921959	0.059130
5	6	0	4.552496	-1.930193	0.114326
6	6	0	5.895240	-1.605279	0.140950
7	6	0	2.998673	1.536766	-0.029424
8	6	0	2.154489	-1.318491	0.033057
9	6	0	1.175630	-0.216915	-0.031922
10	6	0	1.608923	1.178429	-0.060869
11	6	0	0.600163	2.209309	-0.127790
12	1	0	0.945739	3.234422	-0.172127
13	6	0	-0.709325	1.896691	-0.146255
14	6	0	-1.164626	0.501272	-0.108664
15	6	0	-0.156496	-0.509037	-0.060900
16	1	0	7.344123	-0.009339	0.132983
17	1	0	5.624985	1.788501	0.035610
18	1	0	4.216150	-2.959851	0.135665
19	1	0	6.642487	-2.388734	0.184867
20	1	0	-0.416773	-1.559356	-0.013950
21	8	0	1.800524	-2.489643	0.059617
22	8	0	3.351521	2.728477	-0.050665
23	1	0	-1.437157	2.691358	-0.228113
24	6	0	-2.557845	0.173084	-0.112544
25	6	0	-3.555819	1.161602	0.101843
26	6	0	-3.011211	-1.157837	-0.328255
27	6	0	-4.898312	0.842204	0.105768
28	6	0	-4.353658	-1.464117	-0.324852
29	1	0	-2.299376	-1.949816	-0.516021
30	6	0	-5.321046	-0.473174	-0.108723
31	1	0	-5.641254	1.612312	0.276582
32	1	0	-4.666935	-2.489231	-0.493858
33	1	0	-3.272078	2.186741	0.294115
34	6	0	-6.790134	-0.816497	-0.146966
35	1	0	-7.148500	-0.749250	-1.178408
36	1	0	-6.931895	-1.854514	0.177352
37	8	0	-7.594546	0.073012	0.594656
38	1	0	-7.376378	-0.015485	1.526632

para of **5** (1 3)

Center Number	Atomic Number	Atomic Type	Coordinates (Angstroms) X	Y	Z
1	6	0	-6.271778	-0.293743	-0.112007
2	6	0	-5.338952	0.716019	-0.040728
3	6	0	-3.955553	0.418647	-0.014005
4	6	0	-3.556692	-0.944398	-0.064454
5	6	0	-4.519816	-1.951896	-0.136095
6	6	0	-5.865855	-1.636433	-0.160700
7	6	0	-2.965548	1.438822	0.061278
8	6	0	-2.132694	-1.332733	-0.043716
9	6	0	-1.150750	-0.225720	0.030869
10	6	0	-1.568962	1.127611	0.080369
11	6	0	-0.589712	2.156652	0.149662
12	1	0	-0.913394	3.187143	0.207730
13	6	0	0.737706	1.842522	0.147486
14	6	0	1.183257	0.477882	0.098954
15	6	0	0.197296	-0.531183	0.051503
16	1	0	-7.326489	-0.048441	-0.131648
17	1	0	-5.699876	1.738550	-0.006585
18	1	0	-4.178004	-2.979279	-0.173158
19	1	0	-6.608466	-2.422335	-0.219270
20	1	0	0.453814	-1.581803	-0.013895
21	8	0	-1.769015	-2.493872	-0.082921
22	8	0	-3.250652	2.741731	0.114362
23	1	0	1.459267	2.644459	0.227864
24	6	0	2.579993	0.151292	0.084800
25	6	0	3.567720	1.126348	-0.262976
26	6	0	3.038131	-1.167289	0.410487
27	6	0	4.899974	0.810663	-0.297181
28	6	0	4.368836	-1.471706	0.387957
29	1	0	2.327820	-1.924191	0.714197
30	6	0	5.326937	-0.492800	0.029768
31	1	0	5.643082	1.544624	-0.580354
32	1	0	4.701607	-2.469018	0.654840
33	1	0	3.260489	2.123204	-0.547846
34	6	0	6.773756	-0.860358	0.004075
35	1	0	7.042966	-1.248134	0.998577
36	1	0	6.897202	-1.691094	-0.707751
37	8	0	7.535860	0.257935	-0.351161
38	1	0	8.465396	0.021625	-0.402261
39	1	0	-4.198739	2.909147	0.105259

meta of **5** (1 3)

Center Number	Atomic Number	Atomic Type	Coordinates (Angstroms) X	Y	Z
1	6	0	6.279817	-0.202244	0.131479
2	6	0	5.322633	0.790268	0.042158
3	6	0	3.964342	0.464760	0.012188
4	6	0	3.557271	-0.892698	0.072550
5	6	0	4.554527	-1.889919	0.165804
6	6	0	5.888719	-1.547489	0.194876
7	6	0	2.984011	1.560923	-0.079077
8	6	0	2.165400	-1.209917	0.040000
9	6	0	1.173081	-0.196023	-0.044741
10	6	0	1.560403	1.177696	-0.098631
11	6	0	0.579030	2.189860	-0.170578
12	1	0	0.921570	3.216022	-0.230847
13	6	0	-0.748506	1.876165	-0.168277
14	6	0	-1.171940	0.500452	-0.119290
15	6	0	-0.188035	-0.511132	-0.070700
16	1	0	7.331314	0.055283	0.154482
17	1	0	5.596382	1.837394	-0.005894
18	1	0	4.300808	-2.943434	0.220344
19	1	0	6.639137	-2.324866	0.269563
20	1	0	-0.471463	-1.552151	0.000638
21	8	0	1.700625	-2.464945	0.084627
22	8	0	3.307688	2.737623	-0.131802
23	1	0	-1.482479	2.664692	-0.256920
24	6	0	-2.566524	0.163686	-0.103102
25	6	0	-3.545007	1.120785	0.297829
26	6	0	-3.023505	-1.137531	-0.476952
27	6	0	-4.878344	0.799541	0.343445
28	6	0	-4.356316	-1.443519	-0.454731
29	1	0	-2.317137	-1.878167	-0.827537
30	6	0	-5.306107	-0.484589	-0.038096
31	1	0	-5.616645	1.517865	0.674713
32	1	0	-4.692863	-2.426724	-0.765267
33	1	0	-3.232669	2.104417	0.621317
34	6	0	-6.754989	-0.852634	-0.014207
35	1	0	-7.042482	-1.154918	-1.032569
36	1	0	-6.869094	-1.737728	0.629528
37	8	0	-7.505519	0.235598	0.445312
38	1	0	-8.438684	0.008828	0.464927
39	1	0	2.412184	-3.111346	0.126744

3 (0 1)

Center Number	Atomic Number	Atomic Type	Coordinates (Angstroms) X	Y	Z
1	6	0	6.222679	-0.318359	0.001435
2	6	0	5.280581	0.696513	0.000991
3	6	0	3.911739	0.388253	0.000345
4	6	0	3.516036	-0.962523	0.000182
5	6	0	4.476962	-1.973043	0.000642
6	6	0	5.826208	-1.657901	0.001258
7	6	0	2.912523	1.441997	-0.000181
8	6	0	2.078937	-1.333553	-0.000332
9	6	0	1.110356	-0.203775	-0.000751
10	6	0	1.572353	1.171447	-0.000927
11	6	0	0.542214	2.184017	-0.001897
12	1	0	0.807511	3.237075	-0.003056
13	6	0	-0.772757	1.873465	-0.001788
14	6	0	-1.246071	0.503853	-0.001027
15	6	0	-0.216807	-0.498095	-0.000821
16	1	0	7.277325	-0.068252	0.001940
17	1	0	5.588002	1.733882	0.001159
18	1	0	4.133217	-3.000638	0.000528
19	1	0	6.569723	-2.445664	0.001629
20	1	0	-0.463843	-1.551643	-0.000335
21	8	0	1.718914	-2.496768	-0.000390
22	8	0	3.435007	2.695852	0.000087
23	1	0	-1.482259	2.688722	-0.002834
24	6	0	-2.603089	0.170331	-0.000599
25	6	0	-3.643500	1.178566	0.001383
26	6	0	-3.070007	-1.200830	-0.001888
27	6	0	-4.958907	0.870185	0.001876
28	6	0	-4.382208	-1.516050	-0.001568
29	1	0	-2.353094	-2.009083	-0.003530
30	6	0	-5.414254	-0.502344	0.000276
31	1	0	-5.673737	1.687959	0.003720
32	1	0	-4.686742	-2.557538	-0.002796
33	1	0	-3.371124	2.224616	0.002986
34	6	0	-6.721038	-0.871156	0.000432
35	1	0	-7.013706	-1.914610	-0.000786
36	8	0	-7.788956	-0.059896	0.002055
37	1	0	-7.517332	0.863510	0.003407
38	1	0	2.734655	3.352180	0.001706

para of **3** (0 3)

Center Number	Atomic Number	Atomic Type	Coordinates (Angstroms) X	Y	Z
1	6	0	6.239882	-0.304336	0.134299
2	6	0	5.309206	0.704872	0.028077
3	6	0	3.929153	0.397584	0.006850
4	6	0	3.523901	-0.954485	0.098502
5	6	0	4.488771	-1.961645	0.205661
6	6	0	5.833519	-1.646113	0.223801
7	6	0	2.949240	1.423148	-0.101070
8	6	0	2.096561	-1.324465	0.090949
9	6	0	1.124882	-0.214085	-0.018471
10	6	0	1.554555	1.131380	-0.122645
11	6	0	0.563225	2.133366	-0.247679
12	1	0	0.827806	3.178548	-0.373344
13	6	0	-0.773245	1.810856	-0.242686
14	6	0	-1.205944	0.473838	-0.114725
15	6	0	-0.238336	-0.516705	-0.011885
16	1	0	7.295686	-0.060415	0.149934
17	1	0	5.619474	1.739352	-0.039514
18	1	0	4.143727	-2.986268	0.275576
19	1	0	6.575528	-2.430677	0.309589
20	1	0	-0.515045	-1.559034	0.100721
21	8	0	1.729368	-2.489138	0.168642
22	8	0	3.417921	2.684229	-0.179113
23	1	0	-1.509593	2.596581	-0.367887
24	6	0	-2.648589	0.152938	-0.097048
25	6	0	-3.565082	0.996239	0.543676
26	6	0	-3.143690	-1.009211	-0.714807
27	6	0	-4.916499	0.708572	0.571249
28	6	0	-4.487191	-1.307700	-0.700716
29	1	0	-2.456526	-1.670633	-1.230535
30	6	0	-5.424608	-0.455477	-0.058952
31	1	0	-5.571219	1.390008	1.105385
32	1	0	-4.846573	-2.203818	-1.194648
33	1	0	-3.205101	1.880942	1.057573
34	6	0	-6.788333	-0.795420	-0.075590
35	1	0	-7.151004	-1.702028	-0.538695
36	8	0	-7.773868	-0.058489	0.470780
37	1	0	-7.421308	0.755320	0.842848
38	1	0	2.698517	3.320389	-0.160224

meta of **3** (0 3)

Center Number	Atomic Number	Atomic Type	Coordinates (Angstroms) X	Y	Z
1	6	0	-6.252762	-0.241255	-0.130269
2	6	0	-5.317645	0.763896	-0.024584
3	6	0	-3.938948	0.450565	-0.003567
4	6	0	-3.539651	-0.903298	-0.094871
5	6	0	-4.508953	-1.906251	-0.201487
6	6	0	-5.852308	-1.584830	-0.219427
7	6	0	-2.954522	1.471856	0.103800
8	6	0	-2.113945	-1.279533	-0.087519
9	6	0	-1.137381	-0.173390	0.021322
10	6	0	-1.561125	1.173980	0.125156
11	6	0	-0.565382	2.171649	0.249642
12	1	0	-0.825348	3.218022	0.375028
13	6	0	0.769660	1.843279	0.244454
14	6	0	1.196462	0.504335	0.116829
15	6	0	0.224495	-0.481988	0.014528
16	1	0	-7.307489	0.007290	-0.145746
17	1	0	-5.623357	1.799749	0.042741
18	1	0	-4.168422	-2.932400	-0.271147
19	1	0	-6.597770	-2.366160	-0.304794
20	1	0	0.496605	-1.525557	-0.097802
21	8	0	-1.751882	-2.445830	-0.164917
22	8	0	-3.417650	2.735005	0.181539
23	1	0	1.509475	2.625807	0.369234
24	6	0	2.637681	0.177105	0.098932
25	6	0	3.557719	1.016170	-0.542271
26	6	0	3.127820	-0.987005	0.716957
27	6	0	4.907855	0.722570	-0.570054
28	6	0	4.469996	-1.291388	0.702661
29	1	0	2.437878	-1.645240	1.233054
30	6	0	5.410996	-0.443493	0.060410
31	1	0	5.565437	1.400955	-1.104559
32	1	0	4.825556	-2.188914	1.196803
33	1	0	3.201506	1.902278	-1.056374
34	6	0	6.773221	-0.789408	0.076852
35	1	0	7.132017	-1.697448	0.540170
36	8	0	7.761855	-0.056984	-0.469979
37	1	0	7.412784	0.758243	-0.842231
38	1	0	-0.797179	-2.495551	-0.252498

4 (0 1)

Center Number	Atomic Number	Atomic type	Coordinates (Angstroms) X	Y	Z
1	6	0	0.0000000	0.0000000	0.0000000
2	6	0	0.0000000	0.0000000	1.3847433
3	6	0	1.2129401	0.0000000	2.0901009
4	6	0	2.4219378	0.0000244	1.3693365
5	6	0	2.4051780	0.0000315	-0.0250267
6	6	0	1.2019176	0.0000147	-0.7120085
7	6	0	1.2283555	-0.0000551	3.5421930
8	6	0	3.7276082	0.0001886	2.0751282
9	6	0	3.6688421	0.0002260	3.5621030
10	6	0	2.3946264	-0.0001686	4.2556814
11	6	0	2.4607175	-0.0006531	5.6986356
12	1	0	1.5498441	-0.0019378	6.2899249
13	6	0	3.6357350	0.0000765	6.3656625
14	6	0	4.9144280	0.0010615	5.6838949
15	6	0	4.8417518	0.0007824	4.2493210
16	1	0	-0.9431034	0.0000073	-0.5342171
17	1	0	-0.9310725	0.0000194	1.9358750
18	1	0	3.3562225	0.0000802	-0.5442825
19	1	0	1.1929465	0.0000353	-1.7952005
20	1	0	5.7395754	0.0013854	3.6452492
21	8	0	4.7828506	0.0003010	1.4675518
22	8	0	-0.0076201	-0.0000342	4.1056701
23	1	0	3.6010730	-0.0006351	7.4458644
24	6	0	6.1358888	0.0021299	6.3626934
25	6	0	6.2124565	0.0046023	7.8094581
26	6	0	7.4109487	0.0010619	5.6754398
27	6	0	7.3863163	0.0057161	8.4783726
28	6	0	8.5871118	0.0020012	6.3371609
29	1	0	7.4354150	-0.0009179	4.5953299
30	6	0	8.6538267	0.0043316	7.7822442
31	1	0	7.3538458	0.0078970	9.5640431
32	1	0	9.5188711	0.0009174	5.7810466
33	1	0	5.3011618	0.0060763	8.3907942
34	6	0	9.8624806	0.0051044	8.4010026
35	1	0	10.7868809	0.0040250	7.8353745
36	8	0	10.0932184	0.0072308	9.7221195
37	1	0	0.0591370	0.0019149	5.0631677
38	1	0	4.6085504	-0.0000906	0.5235077
